# Chronic lymphocytic leukemia skin infiltration mimicking an ICD pocket infection: a case report

**DOI:** 10.1186/s12872-017-0522-5

**Published:** 2017-03-24

**Authors:** M. Snorek, A. Bulava, I. Vonke

**Affiliations:** 1 Department of Cardiology, Ceske Budejovice Hospital, Ceske Budejovice, Czech Republic; 2 Faculty of Health and Social Sciences, University of South Bohemia, Ceske Budejovice, Czech Republic; 3 Faculty of Medicine and Dentistry, Palacky University, Olomouc, Czech Republic; 4 Department of Hematology, Ceske Budejovice Hospital, Ceske Budejovice, Czech Republic

**Keywords:** Chronic lymphocytic leukemia, Implantable cardioverter-defibrillator, Pocket infection

## Abstract

**Background:**

We are presenting a case report on an unreported and unusual cutaneous manifestation of chronic lymphocytic leukemia in a patient with an implantable cardioverter-defibrillator (ICD).

**Case presentation:**

A 65-year-old man with a history of chronic lymphocytic leukemia (CLL), previously treated with chlorambucil, was referred in October 2013 for extraction of a single chamber ICD due to a suspected device-related infection in the pulse generator area (left-hand side of Fig. 1). The ICD system (Current VR, St. Jude Medical, USA) had been implanted in November 2009. The patient complained of painless erythema with pruritus in the pocket area. Inflammatory blood parameters were C-reactive protein 17.3 mg/L and leucocytes 29.0 × 10^9^/L. Due to the atypical appearance of the pocket area we did not extract the device. Instead, we created an exploratory excision in the skin induration, which had been present for approximately 6 weeks, and conducted a microbiological and histological examination. All cultivation examinations were negative. However, we did histologically show skin infiltration by CD-5 positive low-grade B-cell chronic lymphocytic leukemia/small lymphocytic lymphoma (B-CLL/SLL). Re-initiation of chemotherapy was not necessary and the skin induration completely disappeared within 2 months (right-hand side of Fig. 1).

**Conclusions:**

Complete removal of an ICD system carries considerable risk. In patients with a history of hematological disease, it is crucial to exclude cutaneous manifestations of the disease prior to device removal.

## Background

We are presenting a case report on an unreported and unusual cutaneous manifestation of a chronic lymphocytic leukemia in a patient with an implantable cardioverter-defibrillator.

## Case presentation

**Fig. 1:**
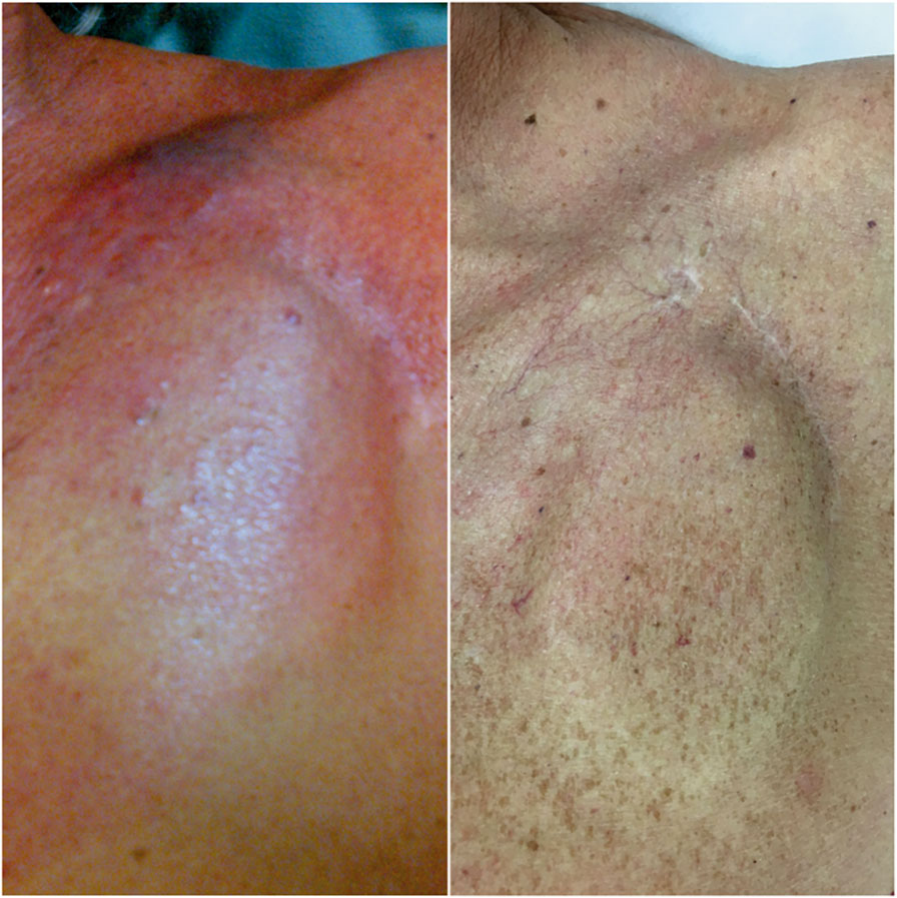
Left-hand side: Painless erythema and mild induration over the device pocket suspicious of device-related infection. Right-hand side: skin induration spontaneously disappeared over following two months

A 65-year-old man with a history of chronic lymphocytic leukemia (CLL), previously treated with chlorambucil, but currently not on active treatment, was referred in October 2013 for extraction of a single chamber implantable cardioverter-defibrillator (ICD) because of a suspected device-related infection in the pulse generator area (left-hand side of Fig. 1). The ICD system (Current VR, St. Jude Medical, USA) had been implanted in November 2009 for primary prevention of sudden cardiac death in the setting of idiopathic dilated cardiomyopathy with low left ventricular ejection fraction (25%). The patient complained of painless erythema with intense pruritus in the pocket area. Inflammatory blood parameters were C-reactive protein 17.3 mg/L (ULN < 5), procalcitonin 0.2 ng/ml (ULN < 0.5) and leucocytes 29.0 × 10^9^/L (ULN < 10), and with 84% lymphocytes. The patient had no fever and his body temperature was in range of 36.5–36.9 °C. Due to the atypical appearance of the pocket area we deferred extracting the device. Instead, we created an exploratory excision of the skin induration, which had been present for approximately 6 weeks, and conducted a microbiological and histological examination. Both aerobic and anaerobic cultivation examinations were negative. However, skin infiltration by CD-5 positive low-grade B-cells was found, which suggested a diagnosis of chronic lymphocytic leukemia/small lymphocytic lymphoma (B-CLL/SLL). Re-initiation of chemotherapy was not necessary because of the disease was considered stable and the skin induration spontaneously and completely disappeared (right-hand side of Fig. 1) over following 2 months and no relapse of symptoms has been observed till present time.

## Discussion

Infectious complications, following cardiac implantable electronic device (CIED) implantation have steadily increased since it first entered clinical practice and are associated with significant mortality. Important host-related risk factors presented in a recently published meta-analysis included: diabetes mellitus, renal disease, chronic obstructive pulmonary disease, corticosteroid use, malignancy, heart failure, and anticoagulant drug use. Procedure-related risk factors included lack of antibiotic prophylaxis, replacement/revision procedures, non-infectious post-operative complications (including dislodged leads and hematoma), temporary pacing, and procedure duration. Average device infection rates have been reported between 1–1.3% [1].

Pacemaker component allergy is a relatively uncommon cause of erythema and pain at the pacemaker implantation site. Diagnosis of a pacemaker component allergy first requires awareness of the problem and then thorough allergy testing with the appropriate allergy test kit provided by the manufacturer. Pacemaker component allergy can present as painful erythema with pruritus covering a large area around the device and may occur months after the implantation procedure. Therapy with a topical corticoid may permanently resolve the skin reaction; otherwise, a more extensively coated or a gold-plated device may need to be implanted in place of the original device [2].

A very rare condition, which can mimic a pacemaker pocket infection, is breast carcinoma. It has been reported in both male and female patients. A firm lesion with signs of skin erosion can be observed near the pacemaker pocket; however, C-reactive protein, white-cell count, and other routine blood tests can be normal [3].

## Conclusions

Removal of CIEDs and all associated leads carries considerable risks. Therefore, in patients with a history of hematological disease, it is crucial to exclude cutaneous manifestations of the disease prior to device removal. Other conditions mimicking pocket infection include breast cancer or pacemaker component allergy.

## Abbreviations

B-CLL: B-cell chronic lymphocytic leukemia
CIED: Cardiac implantable electronic device
CLL: Chronic lymphocytic leukemia
ICD: Implantable cardioverter-defibrillator
SLL: Small lymphocytic lymphoma
ULN: Upper limit of normal
